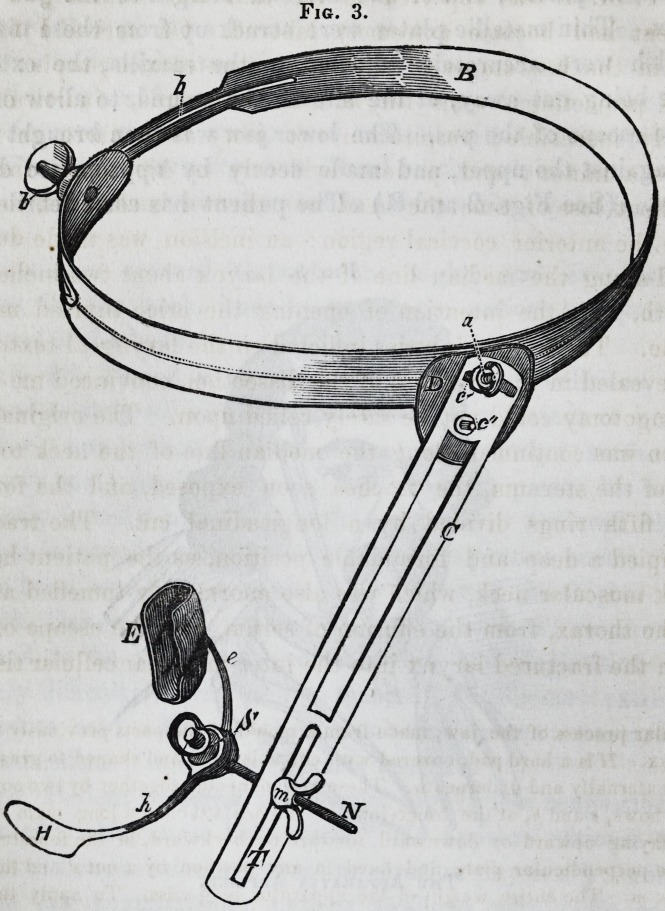# Fracture of the Hyoid and Inferior Maxillary Bones, with Fracture and Dislocation of the Thyroid Cartilages, and Other Injuries; Tracheotomy, &c.; Recovery

**Published:** 1856-04

**Authors:** Albert F. Sawyer

**Affiliations:** San Francisco, California.


### ARTICLE XIV.

*Fracture of the Hyoid and Inferior Maxillary Bones, with Fracture and Dislocation of the Thyroid Cartilages, and other Injuries; Tracheotomy, &c.; Recovery.* (With three wood-cuts.) By ALBERT F. SAWYER, M. D., of San Francisco, California.

A VIGOROUS, muscular man, was at work, July 15, 1854, on a piling machine, which was carelessly overturned while he was near the top, and he fell with it to the ground, a distance of forty-five feet. The iron hammer of the machine, weighing one thousand pounds, was at the time elevated, which, of course, precipitated his descent with the most fearful violence. I saw the patient a few minutes after his fall, and, on examination, found that he had received the following injuries: The body of the lower jaw on the right side, near the symphysis, was extensively comminuted; a large triangular fragment of the maxilla was projecting through a lacerated wound of the integument externally and beneath. A piece of bone above, containing the right canine, and adjoining incisor teeth, was lost at the time the accident occurred. The left angle of the jaw was also fractured, but the separation of the fragments was incomplete. (See Fig. 1.) The extensive bruising of the left side of the head and trunk, indicated that the force of the blow had been received on this part of the body, and as far as the maxilla was concerned, was transmitted to the opposite side, were the comminution existed, on the principle of the arch. The face was frightfully distorted, the chin being greatly displaced to the right side. The cartilages of the larynx were fractured and separated, the right over-riding its fellow. On the left side, the great cornu of the os hyoides could be felt loose and detached from the body of the bone. The neck was much infiltrated with air and serum, and subcutaneous crackling was indicated to the touch over the upper portion of the chest and back. The right radius was broken transversely about three-quarters of an inch above the wrist, the lower fragment being split longitudinally into the cavity of the joint itself. The left patella was much comminuted, the detached fragments of which could be felt and moved about, beneath the integument.

Fig. 1.

The patient was found in a state of great prostration, laboring under the usual signs of concussion of the brain. He could be partially aroused by loud shouting in his ear, or on manipulation about the fractures. Pupils dilated, sluggish to the stimulus of light; respiration slow and measured, without noise; skin bathed with a cold moisture; pulse feeble, cannot be counted. I had him well covered with blankets, external heat constantly applied, and diffusable stimulants, as brandy and ammonia, administered *pro re nata.*

Fig. 1. The Jaw Restored.—1. The large fragment removed at the time of the accident. 2. The piece containing the canine and incisor teeth. 3. The exfoliation subsequently removed ; several smaller exfoliations were also at different times extracted from the wound.

In the course of two hours reaction began to be established, when the patient was transferred to a comfortable bed, and his fractures dressed. Several loose fragments of the maxilla were removed through the external wound, the edges of which were afterwards brought together, close dressings applied, and retained in place by a figure of 8 bandage over the cranium. The fracture of the radius was treated with simple splints; the left lower extremity was flexed on the pelvis, at an angle of thirty degrees, and supported in this position by an inclined plane.

Stimulants ordered through the night, and evaporating lotions for the neck, knee-joint, and the various ecchymosed portions of the body.

*July* 16. Passed a restless night; at times delirious, and inclined to strip off his dressings, followed by periods of heavy slumber; now, when aroused, talks incoherently; surface of the body cool; pupils unequal; head flushed; passes his urine copiously in bed; pulse 110, soft and feeble. Ordered black draught internally, to be followed by a turpentine enema; ice to the head, and blisters to the inner aspect of the thighs.

17*th*. General symptoms of traumatic delirium; respiration inclined to be stridulous; voice husky, and deglutition extremely difficult; bowels well evacuated by medicine; extremities quite cold. Repeat the stimulating enema; mustard to the extremities; injections of warm beef-tea.

18*th*. Patient passed a more quiet night; less congestion of the head; skin moist and warm. He appears rational this A. M., although evincing great prostration physically; wound of the jaw suppurating; patient swallows with more ease; pulse 96, full and soft.

19*th*. Rested well during the night; takes beef-tea by the mouth without difficulty, and with a relish; respiration and voice unchanged; effusion into the knee-joint subsiding. The joint is without much heat or tenderness, and patient makes no complaint of it. Great difficulty is experienced in keeping the fragments of the jaw in coaptation, on account of the comminution of the bone, with its loss of substance, and the frequent change of dressings rendered necessary by the profuse purulent discharge. A mould of the outline of the jaw externally was taken in plaster, and of the alveolar margin of the jaw with wax. Thin metallic plates were struck up from these moulds, which were accurately adjusted to the maxilla, the external one being cut away, at the site of the wound, to allow of the free escape of the pus. The lower jaw was then brought firmly against the upper, and made secure by appropriate dressings. (See Figs. 2 and 3.) The patient has some febrile dis-

Fig. 3.

The Apparatus Applied.

turbance; tongue marked with a white dry centre; pulse 96, with a stong impulse; ordered gruel. ℞.—Infus. sennæ ℥ iv; sulph. magnes. ℥ ss. M. ft. haustus. Pul. Doveri grs. x. Hora somni sumend.

20*th*, 8 A. M. Respiration was somewhat embarrassed during the night; now, however, less dyspnœa than during previous twelve hours; breathing apparently goes on without serious effort. Tumefaction of neck was much increased; swallows

22*

without pain, and craves food; tongue moist; skin of natural temperature; pulse 90, soft and full. Ordered hirud. vj to be

FIG. 3.

APPARATUS SEPARATE.—*B*, a thin ribbon of steel, $1\frac{1}{4}$ inches in width, and long enough to encircle the cranium, covered with chamois skin, with a permanent screw attached to one extremity, playing in the fenestrum *b*, to enable it to be accurately fitted and retained in place by the female screw *b'*. *C* is a thin plate of steel, 6 inches in length, $1\frac{1}{4}$ in width, gently tapering towards the free extremity, and stiffened by a second narrow plate, which is riveted to the first. At *c'* is a fenestrum describing the arc of a circle. This plate is attached to the ear-piece *D* of the strap at the pivot *c''*. It can be moved forward and backward within the limits of the fenestrum, and fixed at any point by the thumbscrew *d*. *E* is a thin silver cap, closing over the molar teeth and applied on each side of the neck, to be followed by fomentations of hot water and spirit.

2 P. M. It was reported to me that the breathing of the patient had become extremely labored. On arrival I found him in the last stages of asphyxia; countenance purple; eyeballs projecting; veins turgid, like whipcords; inarticulate; nearly insensible; respiration not more than three or four times a minute, and patient evidently in his death throes. Without loss of time the head was thrown backwards, to expose the anterior cervical region; an incision was made downward along the median line of the larynx about two inches in length, with the intention of opening the crico-thyroid membrane. The serious injuries inflicted on the laryngeal textures, as revealed in the progress of the dissection, convinced me that laryngotomy could not be safely relied upon. The original incision was continued along the median line of the neck to the top of the sternum, the trachea soon exposed, and the fourth and fifth rings divided by a longitudinal cut. The trachea occupied a deep and formidable position, as the patient had a thick muscular neck, which was also enormously tumefied as far as the thorax, from the effusion of serum, and the escape of air from the fractured larynx into the inter-muscular cellular tissue.

alveolar process of the jaw, made from a mould of the parts previously taken in wax. *H* is a hard pad, covered with chamois skin, and shaped to grasp the jaw externally and underneath. These are connected together by two covered steel bows, *e* and *h*, at the hinge-joint *s*. To *h* is attached a long male screw *N*, playing upward or downward, forward or backward, in the fenestrum *T* of the perpendicular plate, and fixed in any position by a nut *s'* and thumbscrew *m*. The entire weight of the apparatus is ℥ viiss. To apply the apparatus, the head-strap is first fitted, and confined by turning the thumbscrew *b'*, as in Fig. 2. The plate *C* is then swung forward on the pivot *c''*, to bring the fenestrum *T* opposite the angle of the mouth, and fixed by the thumbscrew *d*. The silver cap is fitted over the alveolar margin of the jaw, and the pad adapted inferiorly—the arches *e* and *h* brought together at the joint *s*, their curves just avoiding the integuments of the jaw, and fixed by a thumbscrew. This portion of the apparatus embraces the jaw in a firm grasp. The jaw is then restored to its natural position by drawing it forward and outward, when the long screw *N*, attached to the joint *s*, is inserted into the fenestrum *T*, and fixed there by tightening the thumbscrew *m* upon the nut *s*, the reverse side of the plate.

The patient had ceased to breathe before the trachea was opened. The abundant venous hemorrhage gave considerable annoyance, and I was left entirely without assistance, the nurses being occupied in controlling the wild excitement of a brother of the patient, who, not understanding the purport of the operation, had violently attempted to interfere with it. A quill, which was fortunately at hand, was passed into the trachea. The lungs were then inflated by applying the mouth to the quill, and the wound stuffed with sponge to restrain the oozing. By alternating the inflation by pressure and friction over the chest, by the use of the cold douche, &c., soon satisfactory indications of returning animation and consciousness were obtained. On the first respiratory efforts the quill was removed, and the edges of the tracheal aperture kept asunder as widely as possible, to allow a more perfect ingress and egress of air. A tracheotomy tube was inserted into the trachea as soon as obtained, and tranquil breathing finally restored. About ℥ ss of coagulum and mucus was expelled through the wound and tube. After all hemorrhage had ceased, the upper part of the wound was brought together with the interrupted suture, and the patient left in a comfortable condition. 9 P. M. Respiration free through the tube; patient makes no complaint; chicken tea allowed. ℞.—Spts. nit. eth. ʒj; tr. opii gtt. x; mist. camph. ℥ ss. Hora quaque capiat donec somnis fuerit.

21*st*. Patient slept quietly during the latter part of the night; less tumefaction of the neck; respiration goes on entirely through the tube and the wound. Ordered beef tea and porter, mucilaginous drinks. ℞.—Sul. morph. gr. ⅛; mist. camph. ℥ ss. Hora decubitus.

24*th*. Swelling of neck rapidly passing away; a deep-seated hardness about the larynx and upper portion of the trachea, resulting from the deposit of lymph. Patient has much improved in strength; increased inflammatory excitement about the jaw, without active constitutional symptoms; a clear mucous expectoration through the tube without effort. The dysphagia resulting from the operation has nearly subsided; pulse 85, soft and regular. Ordered a simple enema; asks for and may have the yelk of an egg, and calves' foot jelly.

*28th.* Complains of pains—not constant—at base of lungs; sonorous râles over right anterior chest; cough dry; expectoration thick, viscid, and diminished in amount; subcutaneous emphysema has disappeared; wound of the neck rapidly contracting; tongue covered with a moist gray coat; skin natural; bowels confined; pulse 80, strong and full. A small slough has separated over the fractured patella—disclosing a minute orifice of the size of a probe—evidently communicating with the cavity of the joint, and from which a few drops of synovial fluid has escaped, transforming a simple into a compound fracture. The effusion attending the primary injury has become apparently absorbed, and the joint nearly resembles its fellow. The opening was immediately covered with scraped lint and adhesive strap, and a roller applied from the foot to the groin.

Ordered gruel; black draught ℥ ij. ℞.—Pul. opii c. gr. x; hora somni. ℞.—Syr. ipec. ʒ i; syr. tolut. ʒ iij; acid. hydrocyanic. D. gtt. i, *pro re nata*, sum.

*30th.* Thoracic symptoms relieved; and abundant frothy mucus expelled through the tube; patient complains of a dull ache in the knee joint; dressings removed; some increase of fullness found with tenderness on pressure; external redness and heat; slight synovial discharge from the wound; pain in head; thirst; pulse 80, bounding. ℞.—Hirud. xij around patella, to be followed by warm fomentations. ℞.—Nit. potass. gr. v; tart. antim. et potass. gr. i; sulph. morph. gr. $\frac{1}{8}$ in quaq. tert. hor. sum.

*31st.* General febrile disturbance; knee-joint much swollen; an abundant sero-purulent discharge from the wound; rest much broken on account of pain; bowels freely opened; pulse 86, hard. Apply hirud. vj above and below the joint. ℞.—Pulv. Doveri gr. xv. Hora somni sum.

*August* 1. Less constitutional irritation; knee the same with sanious discharge. Patient rested tolerably through the night. Repeat opiate.

*3d.* Patient makes no complaint; tongue covered with a moist white coat, clean at edges; skin natural; asks for food; pulse 80; a healthy purulent discharge from the joint. An incision was made over inner tuberosity of tibia, and about ℥j of laudable pus evacuated. Beef-tea ordered.

*5th.* 3 A. M. Was informed that the patient was bleeding profusely from the jaw. Instructed the messenger how to make compression on the facial arteries, until I should be able to reach the patient. On my arrival, soon after, I found the patient exsanguine; the skin bathed with a cold moisture. The pulse could scarcely be felt at the wrist. The compression above directed had had the desired effect, and, whilst continued, had perfectly controlled the hemorrhage; on relaxing it, the bleeding was renewed again, evidently poured out from some of the branches of the right facial artery. I cut down upon the facial of this side where it turns over the margin of the jaw, placed a ligature upon it, and the hemorrhage was at once arrested. Ordered bottles of hot water externally; may have hot milk punch *ad libitum.* 4 P. M. A good reaction established; no further hemorrhage. Patient takes nourishment freely; pulse 110. The large coagulum, projecting from the wound externally and internally into the mouth, was carefully removed. The wound has a dry, glazed appearance. Continue stimulants.

*6th.* Patient had a refreshing sleep during the night; considerable swelling over the right side of the jaw, the integument being tense, reddened, and glistening; no suppuration from the jaw; increased vascular excitement about the knee, with a very profuse escape of sanguineous pus from the opening over the patella. Patient has a hoarse dry cough with difficult expectoration; skin of natural temperature, but dry; has a decided appetite; tongue moist; pulse 90, rather sharp. Ordered beef-tea. ℞.—Ol. ricini ℥i; ℞.—Syr. scillæ, syr. ipec., āā ʒss; muc. acac. ℥ss *pro re nata.* ℞.—Pulv. Doveri gr. x. Hor. somn. cap.

*9th.* Patient is mending; wound of the neck rapidly granulating about the tube; less tumefaction of jaw, and suppuration is again established. Left lower limb is much distended with serum above and below the knee. The joint is tender on pressure, but does not give patient much pain.

12*th*. Incision made over outer tuberosity of tibia and about ℥ij of healthy pus evacuated. Patient allowed a free diet. ℞.—Tr. cinchon. c. ʒj; ter in die. Excoriations complained of over the sacrum. Circular air-pillow (caoutchouc life preserver) applied with perfect relief to the patient.

30*th*. A free discharge of pus from wound made to ligate the facial artery; no febrile movement; appetite and digestion good, and the patient is in excellent spirits; pulse 80, of good volume. Fracture of radius examined this A. M. The fragments are found in good position, but only feeble efforts at repair are indicated. Dressings renewed.

*September* 6. An ill-defined tumor of the thigh is observed most prominent at middle of vastus externus muscle; no fluctuation; suppuration has nearly ceased at the knee. Patient has lost all desire for food; nervous system is much depressed; skin hot, dry and harsh to the touch; bowels torpid; tongue marked with a brownish, dry coat; pulse 86, feeble. An incision was cautiously made over the prominent part of the swelling through the extensor muscle, which opened into the cavity of an abscess, evidently communicating with the joint. About ℥iv of laudable pus escaped. A pledget of lint was passed into the wound to prevent the edges from uniting. Since all close dressings on the maxilla were necessarily abandoned after ligature of the artery, the fragments have continued displaced, the chin falling over to the right side, and by their constant irritation were evidently keeping up the suppuration. The probe passed into the wound made to ligate the artery meets with denuded bone, which is undetached and extends towards the place of the fracture. The apparatus figured here (see Figs. 2 and 3,) was contrived to correct this displacement, at the same time not to interfere with the free discharge of matter. The principle consists in presenting an antagonistic force to the powerful muscles attached to the main body of the maxilla, especially those at and near the symphysis, which were retracting it, and acting forcibly on the free extremity of the bone, were dragging it to the opposite side. The short fragment was depressed by the mylo-hyoideus, and overlapped the body of the bone. The fracture at the left angle has become perfectly consolidated. ℞.—Sulph. quin. gr. ij. In quart. hora qua. sum.

13*th.* Patient is much emaciated; general cachexia of the system; complains of pain in the abdomen; abdomen tympanitic; tenderness over the sigmoidfle xure; nervous system irritable; pulse 86, small and sharp. Patient can now wear the fracture apparatus day and night, which could be endured at first only a few minutes at a time. The fragments are retained in the most perfect position by it; and the natural contour of the face restored. The bowels are inclined to be relaxed; continue quinine; apply to abdomen turpentine stupes. ℞.—Tr. opii gtt. xv; eth. chlo. ʒ ss; ol. terebinth. gtt. vj; syr. zinziber. ℥ ss, in quaque hor. repitend donec somn. 2 P. M. Patient has had three hours of refreshing sleep; reports himself as more comfortable; abdominal symptoms much relieved; pulse the same.

16*th.* Typhoid tendencies yielding; all functions of the body well performed; great discharge of healthy pus from the opening at the middle of the thigh, which seems to be the principal outlet for the accumulation within the cavity of the joint. The jaw splint has greatly soothed the irritation resulting from the irregular movements of the fractured bone in the surrounding textures. Suppuration has much diminished; continue quinine; may have a liberal diet with wine.

*October* 17. Patient is slowly convalescing; is kept debilitated from the constant suppuration connected with the knee-joint, on either side of which, occasionally, superficial deposits of pus are evacuated; he has a good appetite; sleep is uninterrupted, and he is free from all fever; circular air-pillows are found of great service in preventing sloughs, where the bony prominences of the body, which is much emaciated, come in contact with the bed; on examination of the fractured radius, firm union was found to have taken place; and all dressings were removed.

*November* 25. General condition of the patient the same as at last report. The wound of the neck has nearly closed around the tube, through which respiration goes on with entire freedom; the tumefaction of the thigh, and the excitement in knee-joint is gradually yielding; the leg was bent at a small angle on the thigh to rest in a favorable position for anchylosis. An incision was made from near the angle of the jaw forward, connecting the two fistulous openings; the edges of the incision dissected up to expose the lower margin and body of the jaw, and a large exfoliation, and several smaller pieces of bone removed. Edges of wound connected by the interrupted suture. Water-dressings.

*January* 2. Patient has been gaining daily in flesh and strength; is now exhibiting malarious symptoms, undoubtedly owing to the locality where he is residing; he is now so far improved as to justify a removal to a more elevated and healthful location; wound of the jaw has entirely cicatrized; the knee is fast resuming a natural appearance; manipulation of the limb, as in changing the dressings, causes only trifling uneasiness. During the twenty-four hours, about ℥j of pus finds its way from the opening in the thigh. Ordered low diet. ℞.—Quin. sul. gr. v; quart quaque hor. sum. ℞.—Pil. hyd. No. iij; hor. som. ℞.—Tr. rhei ℥ss; mane primo sumat.

10*th*. Only a small escape of thin serous pus from the knee; the splint removed this A. M., and the limb allowed to rest on a pillow.

14*th*. The patient having so far recovered as to allow of operative proceedings on the larynx, with a view to a restoration of the natural air-passage, and the permanent closure of the aperture in the trachea, sulphuric ether was administered to him by saturating a sponge, and placing it over the silver tube in the trachea, through which inhalation went on without embarrassment. After complete anæsthesia had been produced, an incision was made through the integument, along the median line of the neck, from the hyoid bone to the tracheal orifice. The tracheal tube was withdrawn a short distance, and supported by an assistant, to prevent the hemorrhage passing through it into the windpipe. The subjacent tissues on either side of the median line were then dissected up, leaving the free margin of the right thyroid cartilage exposed in its entire length, which was found to be displaced, and widely overlapping its fellow. The anatomical relations of the parts were much obscured from the injury primarily, and from the induration following the inflammatory action excited by it. The edges of the wound were lifted up, and the interval between the cartilages separated as well as possible without opening into the cavity of the larynx. After a pause of about an hour, to allow of the complete arrest of the hemorrhage, which had been very profuse, and principally venous, the final steps of the operation were completed. The crico-thyroid membrane was opened with a sharp-pointed bistoury. Into the wound thus made, a strong probe-pointed bistoury was inserted, and an attempt made to separate the thyroid cartilages their entire length. An unexpected difficulty presented itself, for there was found to be an extensive ossific deposit between them which required considerable force to overcome. At this period the condition of the patient became critical. The bleeding had been very free, and from the delay occasioned by the presence of the bony deposit, a large amount of blood had escaped into the air-passage, and the patient began to show alarming symptoms of strangulation. With the handle of the scalpel I succeeded in separating the cartilages wide enough to introduce a piece of sponge, which prevented any further flow of blood. By the application of stimulants, &c., a free expectoration of coagula took place through the tube, and soon the patient began to respire more freely. After a short interval the sponge was removed, and the bleeding found to be perfectly arrested. It had been originally intended to separate the left thyroid cartilage from such adhesions as might be found, turn it back upon itself, elevate it, and unite the two at their free margins. This was found to be impracticable, for the bony deposit had gone on to such an extent that the cartilage was extremely firm and unyielding. It had apparently been fractured and bent upon itself, and occupied the cavity of the larynx. It was still further bound down by the almost cartilaginous hardness of the adjoining textures. All attempts at examination of the larynx with bougies, &c., were attended with violent paroxysmal cough and vomiting, which, in the present weakened condition of the patient, forbade as detailed an examination as could have been desired. The tracheotomy tube was inserted about midway between the division of the cartilages, in order to have its dilating influences on the depressed left thyroid. To accomplish this, it was necessary to drop the hand until the overlapping interval was passed, and then to forcibly carry the tube around the edge of the left thyroid, raising the hand toward the median line of the neck, and pressing the tube downward. The tube was inserted at this position to exercise a lever power on the depressed cartilage. The patient respires with ease through the tube. As every effort at cough provokes hemorrhage, the wound was stuffed with sponge; about the tube cold-water dressings applied; milk-punch *ad libitum.* 10, P. M. Reaction well established; neck much swollen; cheeks flushed; complains of excessive weakness, and pain in deglutition; pulse 120, of good volume. The sponge removed from the wound. Ordered gruel, and pulv. Doveri grs. xv.

15*th.* Strong sympathetic fever; neck much tumefied from violence of inflammatory action about the wound; extreme dysphagia; tongue dry; pulse 96, hard. Apply hirud. vj to neck; an enema of senna. ℞.—Pulv. Doveri grs. xij; hora somni.

17*th.* Patient is very comfortable; active interference with the larynx is attended with severe paroxysms of vomiting and coughing. Tube removed from the larynx, and a wedge-shaped piece of prepared sponge is inserted into the wound to prevent adhesion, and to promote its dilatation. A tube has been worn in the tracheal orifice since the operation to guard against accident. Patient swallows without pain; craves food; pulse 86, full and soft. Porter allowed, with generous diet. The wound of the larynx is kept well dilated by the use of the sponge tent. Bougies are daily passed downwards through the larynx into the trachea, and upwards into the posterior fauces. No. 6 can now be used without much difficulty. These constantly provoke violent retching and vomiting, which severely tax the patient for the time being. A tube is inserted here morning and evening, on a small sized bougie as a guide, between the vocal chords, and retained for an hour. But the patient loses all power of articulation when the tube is in place. Respiration goes on easily through it. A gutta percha plug of the size and form of the tracheal tube is preserved in the tracheal orifice, while the tube is in its new position.

*February* 27. Patient is rapidly recovering his health. He can now bear considerable weight on his left limb; knee-joint apparently firm and solid; a few drops of serum only discharged from the fistulous opening. No attempt at union has been made at the fractured jaw. The various textures are, however, firmly consolidated about the fracture, and the patient exhibits no external deformity. Dilating measures still pursued at the larynx. Bougie No. 9 is easily passed in either direction. Respiration goes on as comfortably through laryngeal as through tracheal tube.

*March* 31. Gutta percha bougies of large size, and fashioned for the purpose, worn as much as possible during the day. The wound has a strong disposition to contract. The sponge-tent still worn during the night. Notwithstanding the use of astringents and caustics on the mucous surface of the larynx, there is still a troublesome irritability attending every interference with it. The bougies can be worn for about two hours, when the patient suffers from a severe pain in the temples, and at right angle of jaw, which soon becomes insupportable. Cheeks are flushed; conjunctivæ suffused; nausea and vomiting follow compelling the removal of the bougie.

*May* 27. Wound of the larynx has now united, except at orifice where the bougies find entrance. Notwithstanding the successful dilatation of the larynx to an extent to allow a sufficient amount of air to pass by the natural passage, and to admit of a free respiration through it, yet every attempt to encourage the air to take this direction has failed. On removal of the tubes, and closure of the orifices, no air can be detected from the mouth. Symptoms of suffocation ensue, requiring the speedy renewal of the tube. In articulation, the diaphragm and thoracic muscles are greatly exerted to insure the production of sound—the patient grunting out his words, as it were, without much distinctness, and in a hoarse whisper. Every expedient having been exhausted in the attempt to restore natural respiration, and as it appeared that nothing further could be accomplished to promote this desirable end, it was determined, after consultation, to close the laryngeal opening, and allow the respiration to continue through the artificial opening in the trachea; firstly, because the tube was worn with less discomfort to the patient in this position than in the larynx; secondly, because the patient could make no articulate sound from the upper opening, probably on account of the interference of the tube with the vocal cords; thirdly, because it was not improbable that there might be troublesome contraction of the trachea during the healing process, especially as the wound has been open for so long a period.

*July* 1. General health of the patient is now fully restored. Knee-joint is firmly anchylosed, and patient can walk about without other inconvenience than results from a stiff limb. Respiration goes on entirely through the tracheal tube. The opening in the larynx had a tendency to become fistulous, but on the application of caustic it soon closed by granulation. There has been no attempt at union in the fractured jaw. Patient can masticate his food well, and the face is free from all deformity. Medical attendance is now discontinued.

*Remarks.*—This case affords an example of the wonderful tenacity of the vital principle, sometimes manifested. At the time of the accident, the severity and complication of the patient's injuries forbade any rational idea of his recovery. For the first few days a liberal exhibition of stimulants was required to overcome the severe prostration resulting from the shock to his entire system, occasioned by the fall. There were no evidences of marked inflammatory excitement until the 20th, five days after his fall, and then his symptoms were of an asthenic character, requiring the utmost caution in the use of depletives. On the morning of the 20th, it was seriously contemplated to cut down upon the larynx, but as the patient appeared to respire with sufficient freedom, the idea was dismissed for the time being, and when called upon to operate in the afternoon, the urgency was such as to forbid any elaborate attempt, by a careful dissection, to restore the larynx to its normal condition. The fracture of the tongue bone is a rare accident. I have been unable to find an instance of it, from such sources as I could examine here, unless connected with violent strangulation from a force applied directly to the bone itself. So large a portion of the jaw was lost by fracture, and subsequent exfoliation, that a bony union of the remaining fragments could not be expected. The first apparatus afforded an excellent dressing, and simple fracture of the jaw can be treated by it with advantage. The articulation of the jaw allows such a variety of movement, that it is impossible to apply a bandage to insure perfect immobility. A roller cannot be applied to the healthy jaw, so that the mouth cannot be opened half an inch. Still, sufficient support is given to the fragments to answer in most cases of simple fracture. All that was expected from the jaw-splint afterward contrived was obtained, thereby restoring the contour of the face; quieting the irritation in the surrounding textures from the unrestrained and irregular movements of the bone when acted upon by the muscles attached to them; insuring, as perfectly as possible, their retention in a natural position after nature had completed her reparative efforts by a consolidation of the soft parts about them. I can conceive of no case of fracture of the jaw where there is displacement sufficient to require mechanical support, and especially in compound fractures attended with suppuration, where this splint, or modifications of it to suit special indications, cannot answer all that is required of a splint, at the same time promoting the comfort of the patient; as in this instance the patient, after becoming accustomed to the splint, found it impossible to sleep without it. The silver cap covering the alveolar margin must necessarily be fitted to each particular case; and oftentimes it might be necessary to duplicate the perpendicular plate, for the opposite side, to exercise a retentive force on both fragments.

The fracture of the patella was an interesting feature in this case. It was remarkable that this bone should have been so comminuted without fracture of the long bones entering into the articulation of the knee-joint. The irritation attending the original fracture was so trifling, that there was every indication that the joint would be preserved with a tolerable degree of integrity. About a fortnight after the accident, when the joint had resumed its natural appearance, the separation of a slough, which appeared to be superficial only, opened into the cavity of the joint, and inflammatory symptoms were kindled up, which for weeks threatened the loss of the limb. The exhaustion produced by the constant drain on his system from this source, kept the patient so weakened that nearly six months elapsed before there could be any justifiable attempt to restore the natural air-passage. In operating upon the larynx, it will be observed that sulphuric ether was the agent employed. The inhalation went on without embarrassment to the patient, without exciting cough or sense of strangulation, or stimulating the nervous system, as is not unfrequently the case where anæsthetics are administered under a more favorable combination of circumstances.

Of the anæsthetics, chloroform, chloric ether, and sulphuric ether, the two former—especially chloroform, which is in the most common use—have now a long array of fatal cases attending their exhibition. We believe there is not a single well-attested case on record of fatality from the inhalation of sulphuric ether. This statement is often challenged, but the contradiction really arises from the careless reports of mortality, often in newspapers or otherwise brought before the public, where the general term of ether is used indefinitely for all anæsthetic agents, and the word "etherized" is made synonymous with anæsthesia, or the effects of any of them. This is certainly an important fact for the consideration of medical men, especially since accidents from the inhalation of chloroform have become so accumulated that the propriety of its administration, unless in the most urgent cases, is becoming seriously questioned. Thence, also, the growing substitution of freezing mixtures, as recommended by Arnott, for producing insensibility to pain, in operations involving superficial parts; which, when carried to a sufficient extent to benumb the textures to be incised, from its likelihood to produce sloughs, or suppurating wounds, cannot be esteemed a sound surgical procedure.

There is no doubt that sulphuric ether may produce death, but this is not so likely to happen where an agent is employed which is, as experience demonstrates, more under the control of the surgeon, and less hazardous to the patient. Its perfect safety, with ordinary care, is proved from the circumstance that, although in extensive use in different parts of the country from the date of its application for anæsthetic purposes, yet no mortality has occurred from it. Any one who has been in the habit of seeing both agents administered, chloroform and sulphuric ether—and chloric ether may be regarded as equally dangerous with the former—will be struck with the immediate depression attending the exhibition of chloroform. At the same time, it acts so gently and insidiously that frequently no warning is given of the escape from the waking into the sleeping condition. With sulphuric ether, on the other hand, the various stages of its action on the human body are prolonged, and can be accurately limited by the most inattentive observer; and the period of excitement always discernible, and often violent, immediately preceding the stertor of insensibility, is of great value to the surgeon, as indicative of the actual condition of his patient. Notwithstanding that accidents have so frequently attended the use of chloroform, and very often so in the hands of the most cautious and skillful practitioners, medical men often justify their preference for, and their fancied security in, the administration of it, for the reason that they have never experienced any unfortunate result. But the repeated cases of death from this source in the practice of men as enlightened as themselves in the administration of it, must necessarily awaken doubts in the minds of those who continue to use it, whether they are not constantly exposed to the same mishap. A large number of the fatal instances have occurred in the practice of dentistry. It can be urged that the want of familiarity of dentists with chloroform renders them unsuitable persons to administer it. The objection, however, loses its force when it is considered that the dentist knows as well as the physician the dangerous properties of chloroform; that he applies it sufficiently often to become familiar with the proper mode of administering it—and that he does not require, neither does he seek to produce that state of complete anæsthesia necessary to be secured in order to perform an elaborate dissection, proceeding with his operation when the patient is sensible of what is transpiring.

The common opinion that their employment is made hazardous in every derangement of the thoracic viscera, is certainly a mistaken one. Not only in our own practice, but in the practice of others, a free inhalation of sulphuric ether is often allowed in organic disease of the heart, as an alleviator to pain, and a sedative to produce rest. In inflammatory disorders of the chest, where the powers of life are failing, and stimulants are required, we believe it can be safely made use of, if any necessity arises demanding its quieting effects. Thus, we had a short time since a patient who had a severe attack of pneumonia in both lungs. He was sinkin grapidly with symptoms of typhoid; at times he was quite delirious, and his expectoration was decidedly purulent. He finally was seized with an obstinate hiccough, which continued without interruption for two days, and resisted the usual antispasmodics employed to relieve him. Convinced that his case could not be made more desperate, I determined, after consultation, to administer to him sulphuric ether. After a few inhalations, the hiccough was interrupted, and the patient fell into a gentle slumber, which continued for a considerable interval. From this time he began to improve. He had two or three attacks subsequently, which yielded as readily as before on the application of the remedy.

Mr. Syme, of Edinburgh, in the March number of the *Lancet*, makes some very judicious remarks regarding the exhibition of chloroform, in which he points out the necessity of regarding rather the condition of the respiration than of the circulatory system; to learn the actual condition of the patient during the inhalations. Cases are well known where the inhalation has been suspended, the circulation appearing sufficiently vigorous, and the surgeon engaged in his operation, when, to his dismay, he has found his patient in a state of collapse, from which he was with difficulty, or never restored. The fancied superiority of chloroform over sulphuric ether, has come from a belief that it is more prompt in its action, less disagreeable to the patient to inhale, without the unpleasant consequences sometimes attending the inhalation of sulphuric ether; while it is generally believed, also, that the life of the patient is equally hazarded where either agent is used. Sulphuric ether is often intensely disagreeable to the patient, and sometimes—especially if the will is antagonistic—produces a sense of strangulation which arrests or delays temporarily the respiration, and affords alarm to the attendants. There is no real danger in this asphyxia, and, in most instances, the ether can be continued regardless of it without compromising the condition of the patient. The happy effects of sulphuric ether were well illustrated in the operation on the larynx. Just previous to the final step of the dissection, the patient was placed thoroughly under the influence of it. The laryngeal cartilages were then separated, which was attended with considerable hemorrhage, a portion of which found its way into the bronchia. I had some anxiety lest the exclusion of air from the lungs by the hemorrhage would produce a fatal asphyxia, or immediate suspension of respiration in the insensible state in which he was lying—as I firmly believe would have been the case had chloroform been the anæsthetic used.

The excitement of the nervous system attending the inhalation of sulphuric ether, if any exists, can be easily restrained by attendants, and the ether continued without regard to it. In such cases, the insensibility proceeds more rapidly as the physical efforts of the patient, in his struggles, compel a full, deep, and rapid respiration. The after effects, as nausea, headache, &c., are not more common than the like results of chloroform, or chloric ether. A difference of a minute or two in time in producing anæsthesia, is a matter of very little consequence. With a pure article of concentrated sulphuric ether, I have invariably succeeded within two or three minutes from the time the sponge was first applied. The ordinary commercial ether is certain in its results; but a little more delay is required to produce the end desired. It has been proved by experience, that up to this time no agent has been discovered for anæsthetic purposes that can be administered with equal security for the patient. In short, it produces perfect insensibility to pain, and never has destroyed life. The trifling objections that may be urged against it fall in comparison with this important consideration; and we believe the day is not remote when we shall see it exclusively used by surgeons for anæsthetic purposes.—*Amer. Jour. of Med. Sci.*

## Figures and Tables

**Fig. 1. f1:**
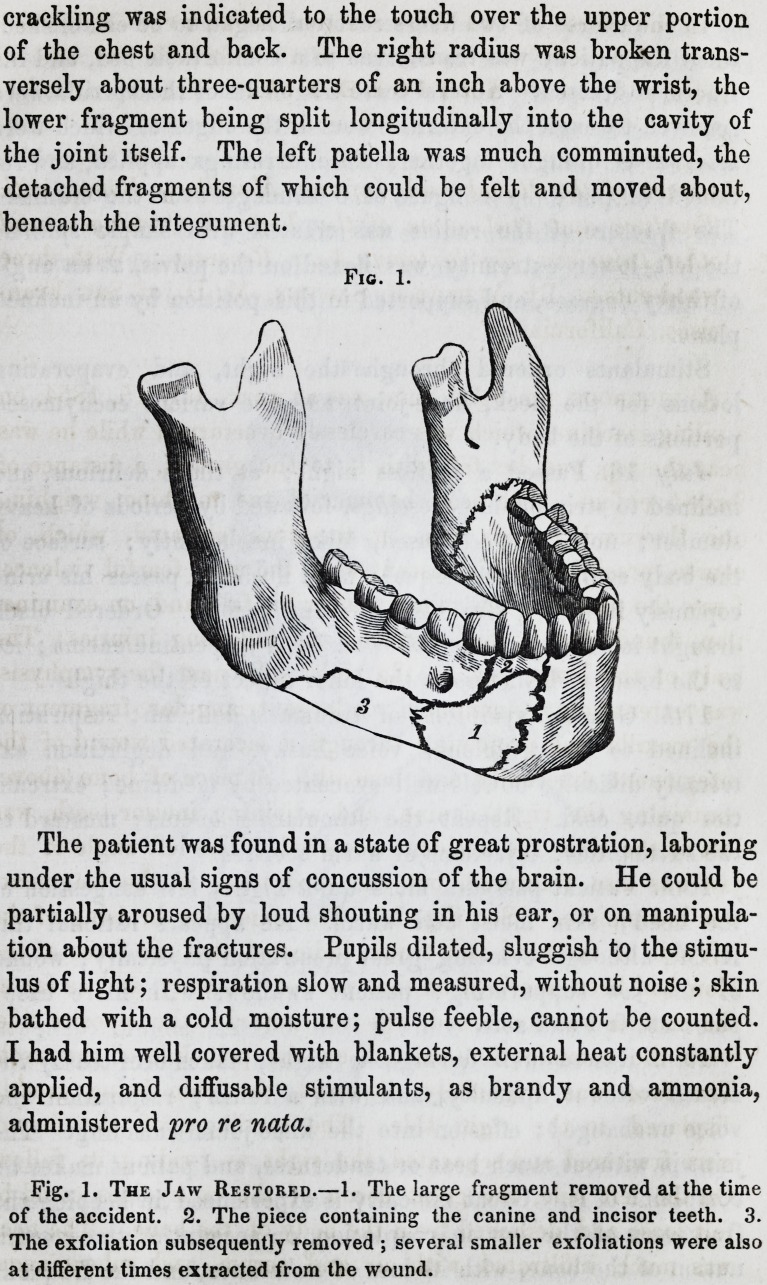


**Fig. 3. f2:**
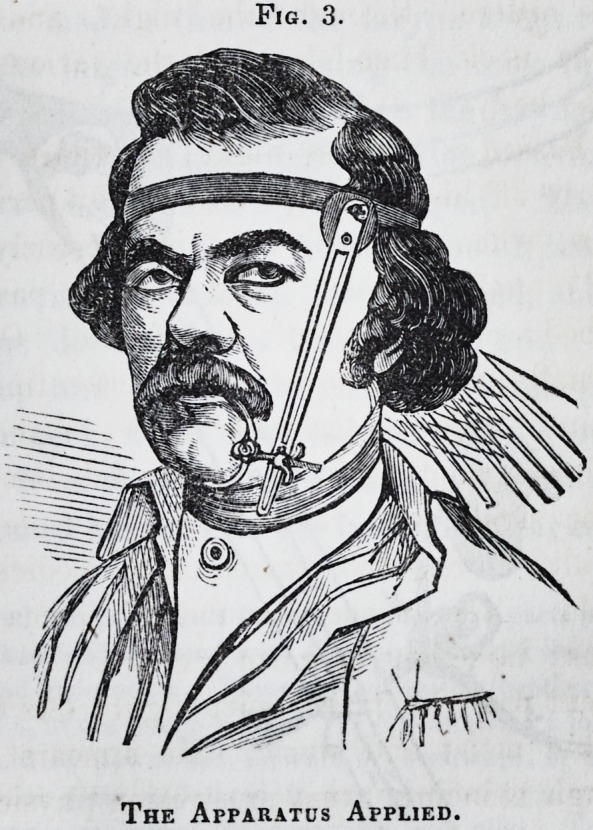


**Fig. 3. f3:**